# Effect of Graphite Nanoplate Morphology on the Dispersion and Physical Properties of Polycarbonate Based Composites

**DOI:** 10.3390/ma10050545

**Published:** 2017-05-18

**Authors:** Michael Thomas Müller, Konrad Hilarius, Marco Liebscher, Dirk Lellinger, Ingo Alig, Petra Pötschke

**Affiliations:** 1 Leibniz Institute of Polymer Research Dresden (IPF), Hohe Str. 6, 01069 Dresden, Germany; mueller-michael@ipfdd.de; 2 Fraunhofer Institute for Structural Durability and System Reliability (LBF), Schlossgartenstraße 6, 64289 Darmstadt, Germany; kh@die-physiker.de (K.H.); dirk.lellinger@lbf.fraunhofer.de (D.L.); Ingo.Alig@lbf.fraunhofer.de (I.A.); 3 Institute of Construction Materials, Technische Universität Dresden (TUD), Georg-Schumann-Straße 7, 01187 Dresden, Germany; marco.liebscher@tu-dresden.de

**Keywords:** polymer-matrix composites (PMCs), graphite nanoplates, electrical, thermal and mechanical properties, melt compounding, dispersion

## Abstract

The influence of the morphology of industrial graphite nanoplate (GNP) materials on their dispersion in polycarbonate (PC) is studied. Three GNP morphology types were identified, namely lamellar, fragmented or compact structure. The dispersion evolution of all GNP types in PC is similar with varying melt temperature, screw speed, or mixing time during melt mixing. Increased shear stress reduces the size of GNP primary structures, whereby the GNP aspect ratio decreases. A significant GNP exfoliation to individual or few graphene layers could not be achieved under the selected melt mixing conditions. The resulting GNP macrodispersion depends on the individual GNP morphology, particle sizes and bulk density and is clearly reflected in the composite’s electrical, thermal, mechanical, and gas barrier properties. Based on a comparison with carbon nanotubes (CNT) and carbon black (CB), CNT are recommended in regard to electrical conductivity, whereas, for thermal conductive or gas barrier application, GNP is preferred.

## 1. Introduction

Graphite [1] is a widely investigated filler which can improve several polymer composite properties (e.g., electrical or thermal conductivity). With micrometer sized stack thicknesses, it has a low aspect ratio, meaning that relatively high contents are needed to obtain enhancement in the electrical and thermal conductivities of polymers. At such filling levels, these property improvements are seriously counteracted by decreased mechanical properties of the final composites, especially deformability and toughness. One way to improve composite properties is the exfoliation of the graphite stacks into thinner graphite flakes. Thereby, a higher filler aspect ratio and a larger specific surface area are expected, which makes exfoliated structures an attractive filler material for composites [2].

In order to generate such graphite nanoplate (GNP) structures—often incorrectly named by their producers as graphene or graphene nanoplatelets—several production routes are available [3]. GNPs can be made by bottom-up approaches such as epitaxial growth via chemical vapor deposition [4,5,6,7] or by the synthesis of graphene based on polycyclic hydrocarbons [8]. Alternatively, top-down approaches such as the chemical reduction of graphite oxide [9,10,11,12], thermal exfoliation and reduction of graphite oxide [11,13,14], un-zipping of carbon nanotubes [15,16] and ultrasonic supported exfoliation of graphite [17,18,19] may be used. Currently, only the top-down methods lead to suitable amounts of GNP materials for industrial use for polymer based composites. There are various ways to exfoliate graphite, including pulverization of expanded graphite, wet ball milling or roll milling [20,21,22]. Most certainly, all these exfoliation approaches result in different GNP morphologies. Due to these differences, the processability of the GNP during melt mixing is different, which is expected to result in various electrical, thermal and mechanical properties of the corresponding composites.

In several recent studies, the influence of the fabrication method and parameters as well as the GNP surface functionality was investigated, mostly for one or two GNP morphology types [23,24,25,26,27,28,29,30]. To use the full potential of the different respective GNP filler materials in polymer based composite applications [31,32,33,34,35,36,37], a more detailed and comprehensive understanding of their structure and processing behavior is needed. Therefore, this study aims to investigate how different GNP morphologies influence their dispersion behavior by melt mixing in small-scale into polycarbonate (PC), and how such differences affect the electrical, thermal and mechanical properties of the composites. Additionally, a comparison to other commonly used carbon fillers such as carbon nanotubes (CNT) and carbon black (CB) is presented in order to evaluate the advantages and disadvantages of each carbon modification. In particular, the shape of the carbon allotropes (spherical, fiber or plate like) plays an important role for their dispersability during the melt mixing process. Furthermore, based on the different shapes, different orientation behaviors are expected in shaped specimens [38,39,40]. In the case of anisotropic arrangement of the filler particles, unique anisotropic properties of the final composite can be generated.

## 2. Materials and Methods

### 2.1. Materials

As amorphous thermoplastic matrix polycarbonate Makrolon PC2600 (Bayer MaterialScience, now Covestro, Leverkusen, Germany) with a density of ρ ≈ 1.2 g∙cm^−^³ (ISO 1183-1) and a glass transition temperature of about *T*_g_ ≈ 148 °C (ISO 11357-1,-2) was chosen. This is a medium viscosity polymer type without any admixtures having a melt volume-flow rate (MVR_300 °C/1.2 kg_) of 12 cm³∙10 min^−1^ (ISO 1133).

A preliminary study was conducted on a variety of about 20 different commercial GNP materials offered by different producers, and three graphite nanoplate powders were selected, which represent different morphology types (Table 1). All of the used commercial GNP materials are according to the manufacturer’s specification non-functionalized. Thus, only physical bonding (such as Van der Waals forces) between GNP and matrix is expected at the interface. Although all of the materials should be classified as “graphite nanoplates” [41], the names used by the corresponding producers are given in that table and are used to distinguish between the materials. For comparison, commercial and typically used carbon nanotubes (CNT) and carbon black (CB) were used as reference carbon materials (Table 1).

### 2.2. Processing

Polycarbonate-carbon filler composites were produced using a small scale co-rotating twin-screw microcompounder (DSM Xplore, Sittard, The Netherlands) with conveying screws and a chamber volume of 15 cm^3^. PC granules and the carbon filler powder were dried in a vacuum oven at 110 °C for at least 4 h. The filler material and the polymer were first dry-blended by slightly shaking in a closed glass vessel, and 13 g of the premixture were fed in the hopper of the compounder. As dry carbon nanomaterials, especially nanotubes, are respirable and may be harmful, they have to be manipulated with care and specific precautions such wearing respiratory protection and working in a fume hood is necessary. The mixing conditions were set to melt temperatures between 240 and 320 °C, with different mixing speeds of 50, 150 and 250 rpm and mixing times between 5 and 30 min. The extruded strands were taken out at the set screw speed and were compression molded at 250 °C, 2.5 min pre-melting, 1.5 min compression at 50 kN into plates or bars (Table 2) using a hot press (Model PW 40 EH, Paul Otto Weber GmbH, Remshalden, Germany). Afterwards they were cooled down quickly to room temperature using a chiller or a hot press (Model PW 20, Paul Otto Weber GmbH, Remshalden, Germany) equipped with a vacuum compression mold where the samples cool down to room temperature by air cooling.

### 2.3. Characterization Methods

#### 2.3.1. Particle Size Distribution of Pristine Carbon Fillers

Carbon powder particle size distributions were determined by laser diffraction using a Helos/BF particle size analyzer coupled with a RODOS dry dispersion unit and ASPIROS microdose module (Sympatec, Clausthal-Zellerfeld, Germany). The measurements were performed at a pressure of 0.5 bar with a lens allowing detection in the range from 0.5 to 875 µm.

#### 2.3.2. Investigation of the Pristine Morphology of Carbon Fillers

Scanning electron microscopy (SEM) was performed on the pristine carbon materials using a Field Emission Electron Microscope (FEG-SEM as well as NEON40, Carl-Zeiss AG, Oberkochen, Germany). The CNT material was deposited in its as received dry state on a double-sided adhesive copper tape and examined using a SE2-detector.

#### 2.3.3. Bulk Density Measurement of Pristine Carbon Fillers

The bulk density of the different carbon materials was determined according to EN 1097-3. A clean and dry stainless steel cylinder with a known volume was filled with the carbon filler powder and weighed three times. The powder excess standing over the cylinder edge was carefully removed. Subsequently the weight of the filled cylinder was determined and the bulk density $\vartheta_{B}$ was calculated using equation:

(1) $$\vartheta_{B}=\frac{m-m_{0}}{V}$$

m is the filled cylinder weight, $m_{0}\;$is the empty cylinder weight and V is the cylinder volume.

#### 2.3.4. Carbon Filler Dispersion

To determine the state of filler macrodispersion in the composites, light transmission microscopy (LM) investigations were performed according to the standard ISO-18553 on thin sections (5 μm thickness) taken along and across the length direction of the extruded strands. An Olympus microscope BH2 combined with a camera DP71 (Olympus Deutschland GmbH, Hamburg, Germany) was used. The area ratio of the carbon filler structures was determined from the LM images using the software ImageJ Version 1.43o by calculating the ratio $A_{r}$ (%) of the area of remaining carbon filler structures $A_{A}$ to the total area of the image $A_{0}$, as shown in Equation (2).

(2) $$A_{r}=\frac{A_{A}}{A_{0}}$$

Whereby, according to the ISO-18553 standard, only structures with circle equivalent diameters >5 μm were regarded. The particle size distributions based on circle equivalent particle diameters are shown with size classes of 5 µm. For its calculation, a plugin was programmed which automatically detects the particles based on contrast differences between the polymer matrix and remaining particle structures. For quantification, 10 cuts were investigated for each sample and the mean value and standard deviation as a measure for the homogeneity are shown in the plots.

To investigate the filler shape at the nanoscale, a transmission electron microscope (TEM, LIBRA-120, Carl-Zeiss GmbH, Oberkochen, Germany) with an acceleration voltage of 120 kV was used. Observations were done on ultra-thin spin coated PC composite samples (spin-cast from chloroform), with a thickness of about 80–100 nm, which were detached from the silicon wafer using sodium hydroxide solution.

#### 2.3.5. Electrical Properties

The electrical resistivity of the as-received carbon filler powders was measured using the PuLeMe (Pulverleitfähigkeitsmessung), instrument developed and constructed at the Leibniz Institute of Polymer Research Dresden, Germany [50,51]. The powder was filled into a cylinder (40 mm length, 5 mm diameter) with a lower fixed gold electrode and a movable upper gold electrode driven by a stepper motor and compressed between the two electrodes up to a pressure of 30 MPa. The electrical volume resistivity of the compressed powders was determined using a Keithley 2001 multimeter (Keithley Instruments Inc, Cleveland, OH, USA).

The electrical volume resistivity of compression molded plates was determined according to the standard ASTM-D257 (ASTM International, West Conshohocken, PA, USA). At least three compression molded samples were measured to obtain the geometric mean value with the associated standard deviation of resistivity. The measurements on the pressed plates were performed through the sample using a Keithley 8009 Resistivity Test Fixture combined with a Keithley electrometer 6517A (both Keithley Instruments Inc., Cleveland, OH, USA).

#### 2.3.6. Mechanical Properties

Tensile properties of neat PC and PC composites were tested on miniature dog bone tensile bars with a gauge length of 16 mm, a width of 4 mm and a thickness of 0.5 mm punched from the compression molded plates. For each formulation, 8 tensile tests were performed. According to DIN 53504/S3a/1, Young’s modulus E_t_, yield stress σ_γ,_ maximum stress σ_M_, stress at break σ_B_, strain at yield point ε_γ_, strain at maximum stress ε_M_ and strain at break ε_B_ were determined. The dynamic mechanical thermal analysis (DMTA) was carried out in rectangular torsion geometry using a ARES G2 (TA Instruments, New Castle, DE, USA) between −30 °C and 200 °C under nitrogen atmosphere at a frequency of 1 Hz (strain 0.2%) and a scan rate of 2 K min^−1^.

#### 2.3.7. Thermal Behavior (DSC)

Differential Scanning Calorimetry (DSC) analysis was carried out using a DSC Q 1000 (TA Instruments, New Castle, DE, USA) between −80 °C and 200 °C under nitrogen atmosphere at a scan rate of 10 K min^−1^. To evaluate the influence of different carbon fillers on the thermal behavior of the PC composites, heating–cooling–heating cycles were performed. The glass transition temperature (*T*_g_) was determined during the second heating.

#### 2.3.8. Thermal Conductivity

The thermal conductivity of the compression molded carbon filler loaded composites was measured using a Hot Disk TPS 500 Thermal Constants device (Hot Disk AB Company, Gothenburg, Sweden) combined with a kapton sensor (Ø 6.403 mm) at 24 °C. The sensor was therefore clamped between two round composite plates (Ø 25 mm; thickness 5 mm) and was heated for 80 s with 0.6 mW. Subsequently, the thermal conductivity was determined by observation of the temperature and the change in the sensor electrical resistance over the time.

#### 2.3.9. Gas Permeability

The oxygen permeability through the composites was measured using a gas permeability test instrument GDP-C (Brugger, Munich, Germany). The vacuum dried round composite plates (Ø 120 mm; thickness 0.2 mm) were placed in the permeation cell, whereby the test sample separates this cell into two sections. To remove the embedded gases in the polymer, both sides of the cell were evacuated for 1 h. Subsequently, one cell section was charged with dry oxygen (flow rate 40 mL∙min^−1^) at 20 °C. The gas permeability was calculated from the pressure increase in the second cell section (known cell volume) as a function of time.

#### 2.3.10. Mixing Energy

Based on the recorded force values of the twin-screw microcompounder during the compounding, the specific mechanical energy (SME) input was calculated according to equation:

(3) $$SME=\frac{2\pi Nr}{m}\int_{t=0}^{t_{i}}Fdt$$

where *N* is the selected screw speed, *r* is the geometric factor of the melt mixing compounder (for DSM15: 0.002 m), *m* is the used composite weight, *F* is the recorded force and *t_i_* is the mixing time.

## 3. Results and Discussion

### 3.1. Morphology of the Filler Materials

**Figure 1:**
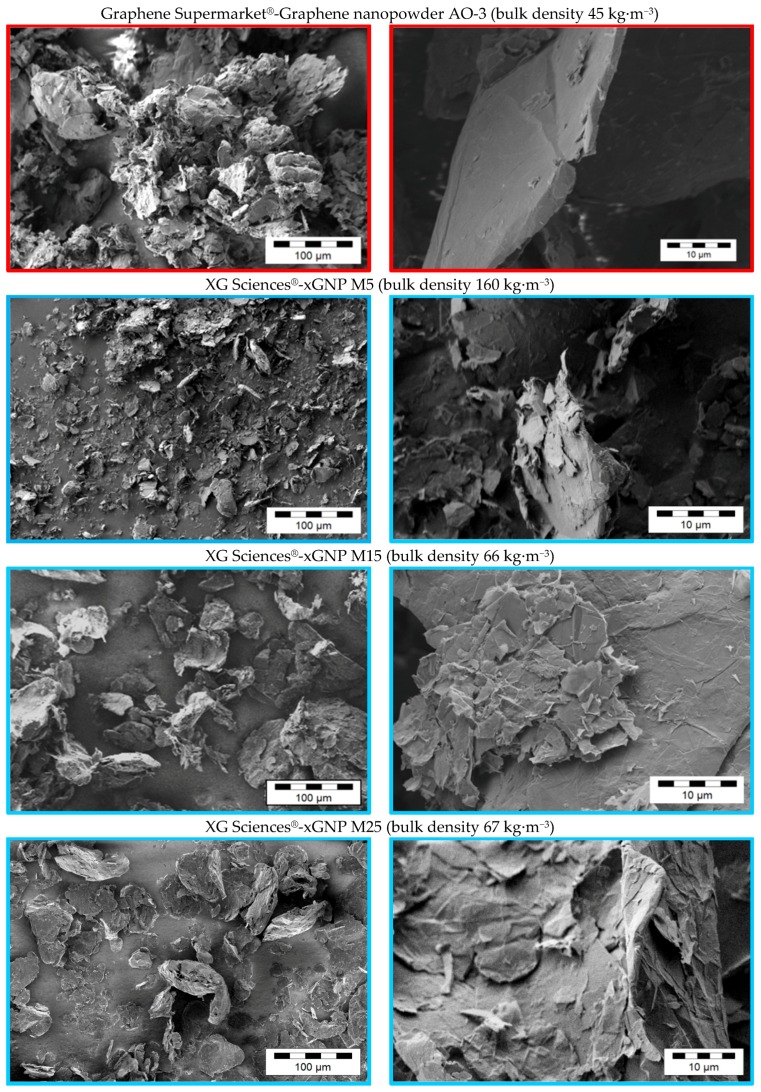
SEM images of different as-received commercially available GNP powders with compact platelet structure.

**Figure 2:**
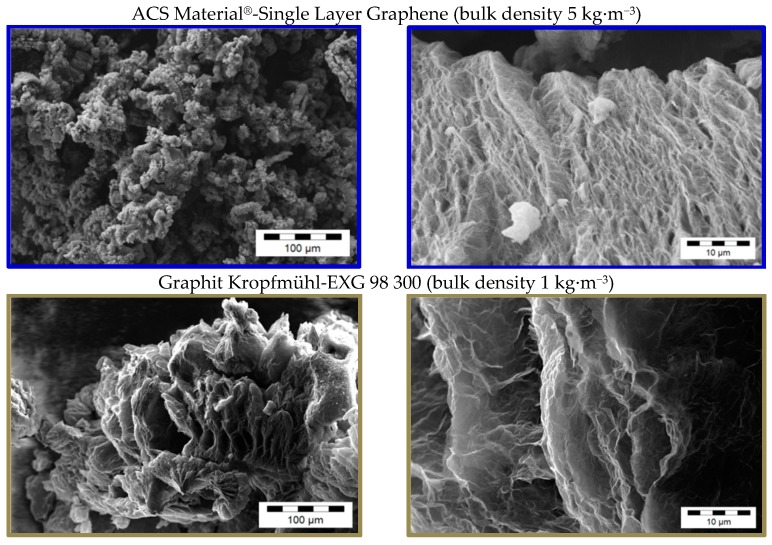
SEM images of different as-received commercially available GNP powders with lamellar worm-like structure.

**Figure 3:**
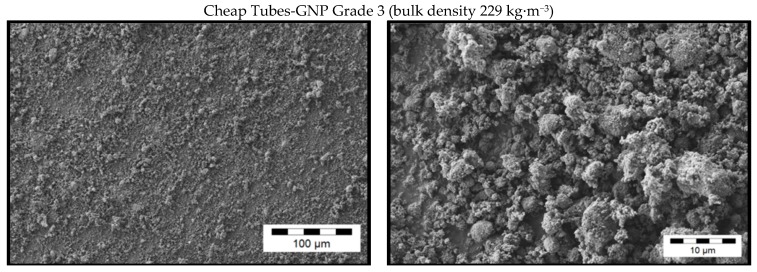
SEM images of as-received commercially available GNP powder with a fragmented plate structure.

**Figure 4:**
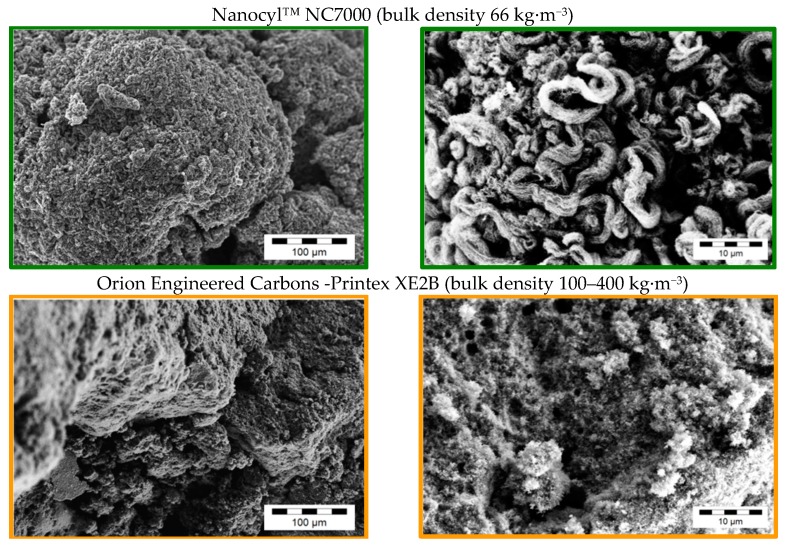
SEM images of as-received commercially available reference carbon filler powders.

Due to the various fabrication methods of the commercial carbon fillers, significant differences in the GNP morphologies can be observed. Figure 1, Figure 2, Figure 3 and Figure 4 show scanning electron microscope (SEM) images of the commercially available GNP fillers as well as the CNT and CB reference materials. The graphene nanopowder AO-3 and XG Sciences xGNP´s exhibit a compact platelet-like structure with a thickness of few micrometers. These materials are produced by the suppliers using a thermal expansion process, likely followed by a mechanical fragmentation process. In comparison, the ACS Material^®^-Single Layer Graphene and Graphit Kropfmühl EXG 98 300 powders exhibit a lamellar worm-like structure. Such a shape is typical for expanded graphite which is thermally reduced. The Cheap Tubes GNP Grade 3 material looks more like a carbon black structure.

**Figure 5:**
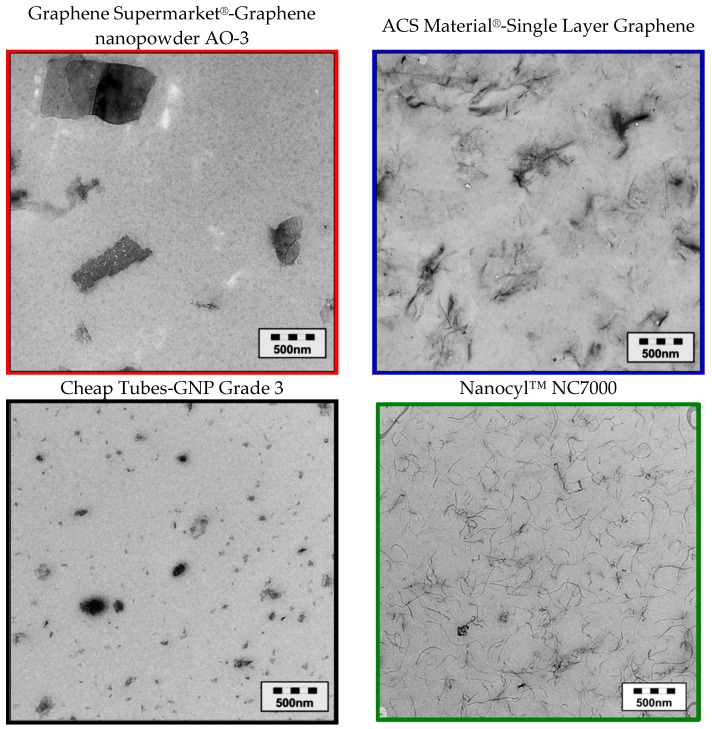

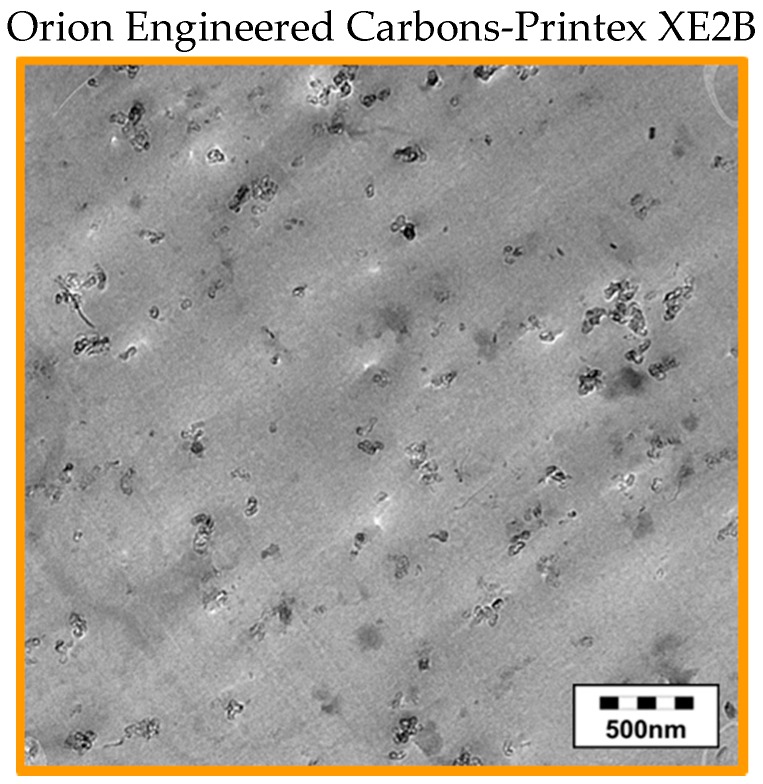
Comparison of the shapes of the different used carbon filler after embedding in polycarbonate (TEM images).

In transmission electron microscopy images (Figure 5), the Cheap Tubes GNP plates appear to be very fragmented and some of them even much smaller than the spherical particles of CB. In contrast, the plate sizes of the graphene nanopowder AO-3 are substantial larger and ACS Material^®^-Single Layer Graphene is comprised of high aspect ratio plates which appear to be wrinkled.

Due to differences in morphology, the different GNP materials have different bulk density values (Table 1). This property plays an important role when feeding these materials into the hopper of the compounding machine. Low bulk densities of the filler result at a given percent mass in large volumes which may not be possible to feed. Remarkably, the ACS Material^®^-Single Layer Graphene and Graphit Kropfmühl EXG 98 300 powders, which exhibit the expanded graphite-like structure, also possess by far the lowest bulk densities of all investigated samples.

### 3.2. Dispersion Behavior of the GNP Fillers

To get a comprehensive understanding about the influence of the different GNP morphologies on their dispersion behavior, the melt mixing processing parameters such as temperature and screw speed were varied for three materials which represent each plate structure type. Changes in the GNP size distribution and dispersion were tracked by the area ratio calculation of the GNP primary structures.

**Figure 6:**
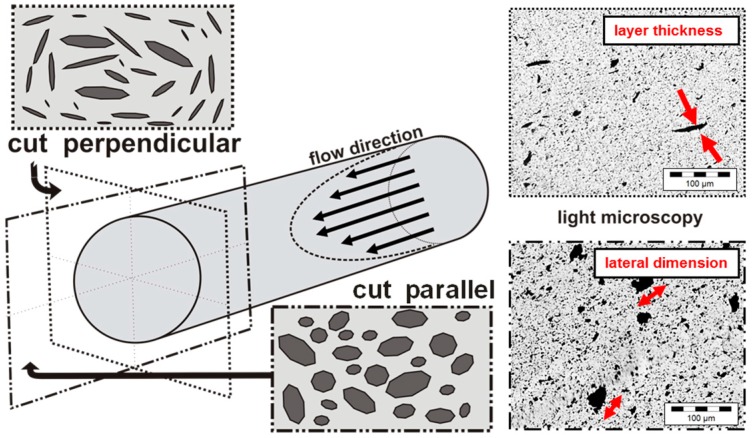
Orientation of graphite platelet structures in extruded strands: (**left**) schematic figure of platelet orientation along strand flow direction by extrusion out of the die; and (**right**) transmission light microscopy pictures of samples cut perpendicular to the strand direction (shows mainly the layer thickness) and cut parallel to the long-axis of the strand (shows the lateral dimension of visible GNP structures), here shown for 1 wt % Graphene nanopowder AO-3 in PC.

**Figure 7:**
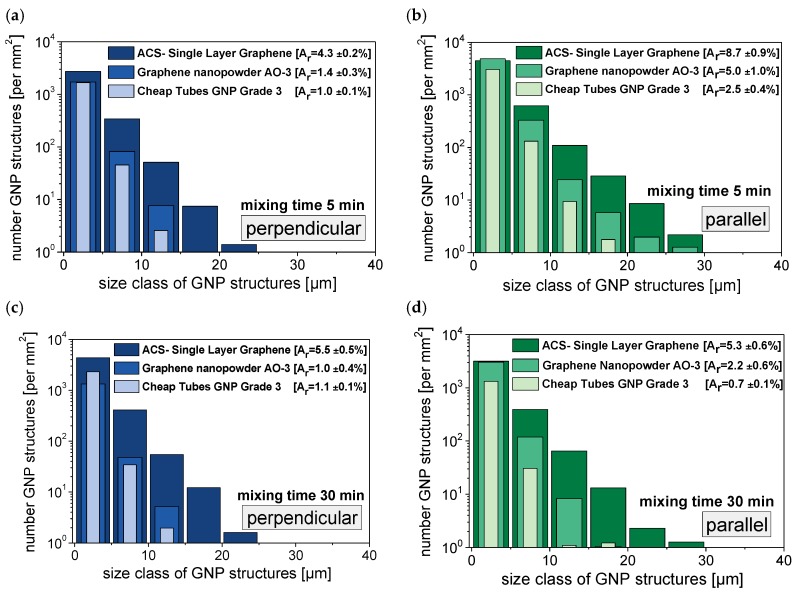
Anisotropy effect in the evaluation of the area fraction of GNP structures (PC with 1 wt % GNP), based on light microscopy images: perpendicular to the strand direction (**a**) after 5 min and (**c**) after 30 min mixing; parallel to the strand direction (**b**) after 5 min and (**d**) after 30 min mixing; particle size distributions based on circle equivalent particle diameters.

**Figure 8:**
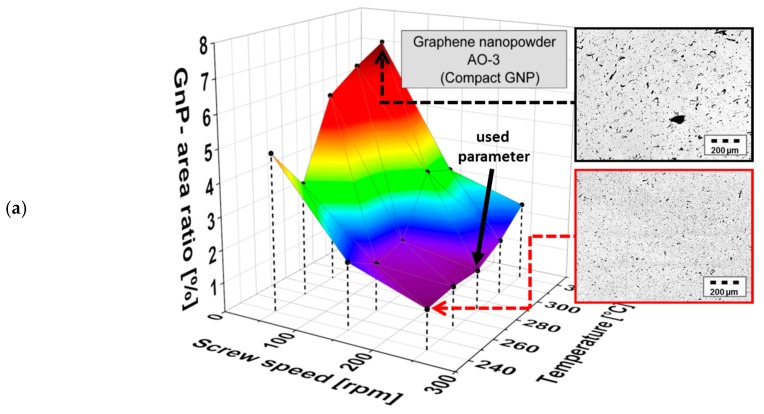

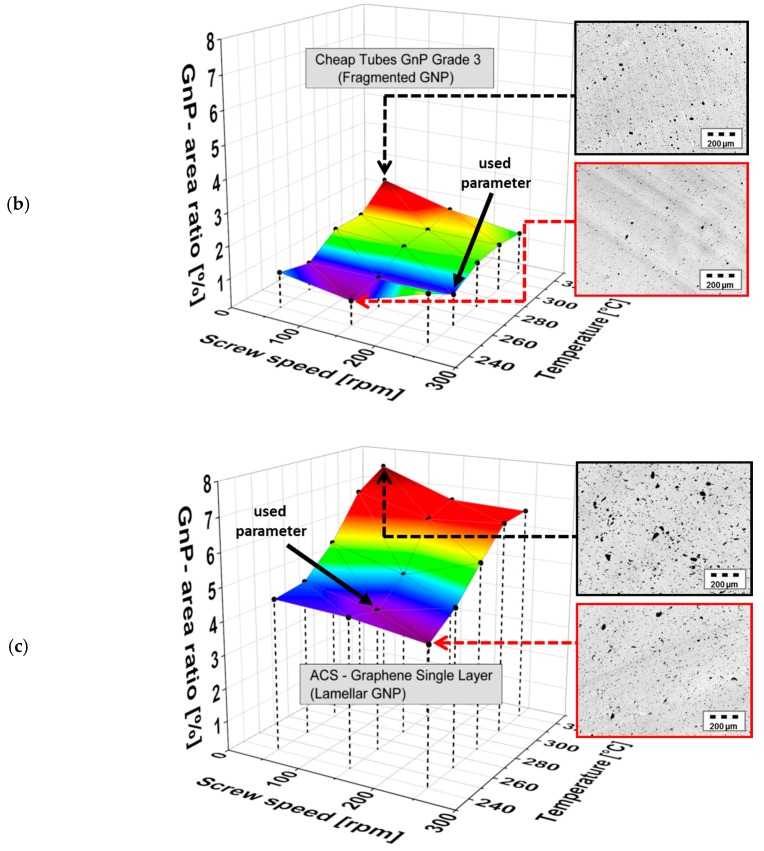
Comparison of the area ratio $A_{r}$ of microscopically visible GNP structures of different GNP morphologies in PC as a function of screw speed and melt temperature (**a**) Graphene nanopowder AO-3, (**b**) Cheap Tubes GNP Grade 3 and (**c**) ACS-Single Layer Graphene (filler content 1 wt %, mixing time 5 min) together with light microscopy images indicating the state of dispersion for selected processing parameters (cuts performed perpendicular to the strand direction).

GNP dispersion was characterized by LM analysis of thin sections of extruded strands. Due to the anisotropy of the GNP primary structures, an orientation takes place during the extrusion of the strands. Thus, different area ratios are detected perpendicular or parallel to the extrusion direction (Figure 6). In perpendicular direction mainly the particle thickness is seen, whereas in the parallel direction mainly the lateral dimension of the GNP structures is seen. Therefore, larger particle sizes are visible in the parallel direction and accordingly this area ratio is higher (Figure 7 and Figure 8). However, when aiming to fully exploit the potential of the graphite nanoplates by exfoliation into thinner structures, this progress can only be seen when images cut perpendicular to the strand direction are studied. Thus, for more detailed investigations, the cutting direction perpendicular to the extrusion direction was selected.

The influence of the melt processing parameters screw speed and temperature at a fixed mixing time of 5 min on the dispersion of the three different GNP morphologies is shown in Figure 8 for 1 wt % filler in PC. All three materials follow the same general trend: better GNP macro dispersion is observed at lower melt temperature and higher screw speed.

Due to the differences in the starting particle sizes of the GNP powders (see Table 1), the absolute changes in the GNP area ratio values differ in the corresponding composites. The GNP area ratios vary between 1.0% and 2.4% for the Cheap Tubes GNP Grade 3 fragmented material, which also had the smallest starting particle sizes and a low layer thickness. For the lamellar ACS-Single Layer Graphene, A_r_ values between 2.4% and 7.0% and a stronger dependence on mixing conditions were found, illustrating that the relatively large expanded structures were susceptible to breaking into thinner ones. However, even at the highest energy input, the material exhibits the largest particle sizes in this comparison. GNP Graphene nanopowder AO-3 material exhibited area ratios between 1.0% and 7.0%, illustrating the largest variation as a function of processing conditions. These results clearly show that independent from GNP primary structure, a complete exfoliation by melt mixing in polycarbonate was not achieved, even when using a large range of processing parameters. The study from Liebscher et al. [26] encountered the same dispersion problem of XGnP M5 in PC/SAN blend systems; by increasing the shear stresses the primary GNP structures could not be fully exfoliated there, too.

**Figure 9:**
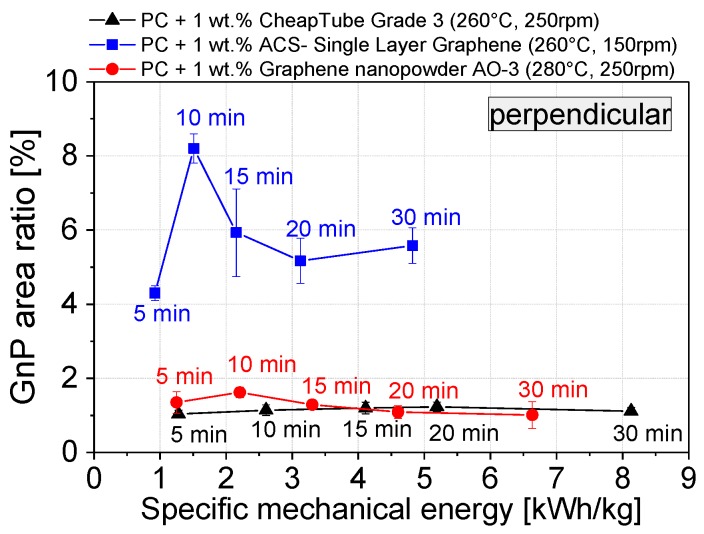
Area ratio of the microscopically visible GNP structures as a function of the specific mechanical energy SME; small numbers indicating the mixing time (cuts performed perpendicular).

Based on the results of the first set, a specified combination of melt temperature and screw speed was selected for each material and a second set of processing was performed. These settings for each material are as follows): ACS-Single Layer Graphene 260 °C, 150 rpm; Cheap Tubes GNP Grade 3 260 °C, 250 rpm; and Graphene nanopowder AO-3 280 °C, 250 rpm. For these conditions, the mixing time was varied between 5 and 30 min, The GNP area ratio vs. the introduced specific mechanical energy (SME) is shown in Figure 9.

Interestingly, already after 5 min mixing time, corresponding to 1.2 kWh∙kg^−1^, for Cheap Tubes GNP Grade 3 and Graphene nanopowder AO-3 relatively constant area ratio values are achieved. In contrast, ACS-Single Layer Graphene has in general much higher values. The area ratio first increases up to 10 min mixing time (1.5 kWh/kg) and then decreases for longer mixing times. Above 3.1 kWh∙kg^−1^ the area value seems to remain constant, however at a much larger level than for the other two materials. The increase in the area ratio of ACS- Single Layer Graphene at a mixing time of 10 min may be related to the further expansion and decomposition of the lamellar wormlike structure (Figure 2). This hypothesis can be supported when considering the number of GNP structures per mm^2^ (Figure 7). This number increases from 3132 at 5 min mixing time to 6467 particles per mm^2^ after 30 min mixing.

If significant exfoliation took place during processing, a continuous decrease of the area ratio would be expected and in case of full exfoliation into graphene no structures should be seen anymore. This was actually not observed.

When comparing the particle sizes measured parallel to the strand direction (Figure 7, right column), a decrease in particle size occurs when the total specific mechanical energy input is increased by prolonging the mixing time from 5 min to 30 min. Both the lateral dimensions of the GNP materials and the GNP aspect ratio decrease. This is an undesired effect as the large plate dimensions and the high aspect ratio are desirable for attaining a low electrical percolation threshold, high mechanical reinforcement and improved barrier properties. The ACS Single Layer Graphene shows the highest numbers of particles per mm^2^, followed by Graphene Nanopowder AO-3 and Cheap Tubes GNP Grade. The latter two graphene materials in particular exhibit a significant decrease in particle size influenced by mixing time. The ACS Single Layer Graphene structures show a decrease in the number of visible structures only in higher particle size classes (over 20 µm). However, in comparison to the other two GNP materials, lower specific mechanical energy (4.9 vs. 6.7 and 8.2 kWh∙kg^−1^) was applied at the selected conditions during 30 min melt mixing (Figure 9).

For a third set of mixing parameters, the GNP concentration was varied to study the GNP effect on different composite properties. Therefore, in respect to balance between GNP dispersion and plate size reduction, the mixing time was fixed to 5 min, and mixing temperature and screw speed were selected for each material separately according to the best state of dispersion. However, to produce highly loaded GNP composites required to achieve a certain electrical conductivity, unprocessable high composite viscosities can be expected when the mixing temperature is too low (240 °C). At this temperature, the best dispersion was achieved in all cases. Therefore, the processing temperature was adjusted to be at or above 260 °C, in order to prevent the overload of the compounder’s engine. The conditions selected for the production of this third set of composites are shown in Table 3.

### 3.3. Electrical Properties

**Figure 10:**
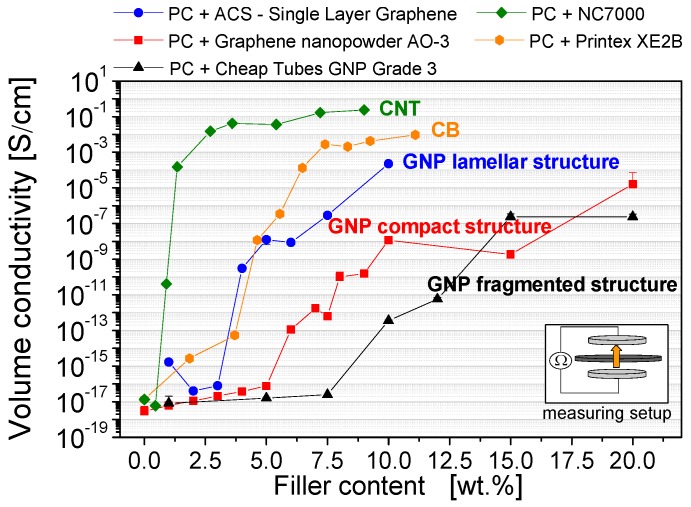
Electrical percolation behavior of various commercially available carbon fillers in polycarbonate (dispersion optimized processing parameters are used), measured through the thickness direction of compression molded plates (Keithley 8009 Resistivity Test Fixture; plate configuration).

The electrical percolation behavior of the composites formed with three different GNP materials is shown in Figure 10. For comparison, the percolation thresholds of carbon black and carbon nanotubes are presented in the same plot. As mentioned before, the selected melt mixing parameters for composite preparation were optimized regarding GNP dispersion and processability (Table 3). However, these mixing parameters are possibly not the favored ones to achieve the highest possible conductivity levels. During the mixing, relative high shear energy was applied to get suitable filler dispersion in the sense of exfoliation, whereby a higher tendency of lateral GNP plate size reduction or CNT length shortening can be noted. The expected resulting reduced aspect ratios lead to a lower statistical possibility of filler contacts and consequently the percolation threshold may be shifted to higher filler contents as compared to when more gentle mixing conditions are used.

For GNP materials, the required filler loading to build up an electrical percolated network ranges is between 4 and 10 wt %. In contrast, the percolation threshold of CNTs—1 wt %—is significantly lower. Among the different GNP filler types, the lowest threshold of 4 wt % can be obtained using the ACS-Single Layer Graphene material, which has a lamellar structure.

The compact GNP structure material (Graphene nanopowder AO-3)—having a threshold between 7 and 10 wt %—and the fragmented structure material (GNP-Cheap Tubes Grade 3)—with a threshold between 10 and 15 wt %—do not increase the electrical conductivity sufficiently at suitable loadings. Interestingly, at higher loadings the conductivities of the GNP composites did not achieved the same values as the CNT or CB filled composites. One reason for the differences in conductivity might be the orientation of the GNP during the compression molding, which is orthogonal to the electrical conductivity measurement direction. The respective differences in the percolation behavior between the GNP types are mainly based on the GNP morphological differences (Figure 2 and Figure 5, Table 1). As a result of these, different plate aspect ratios and different dispersion behaviors during the melt mixing process are obtained (Figure 8).

**Figure 11:**
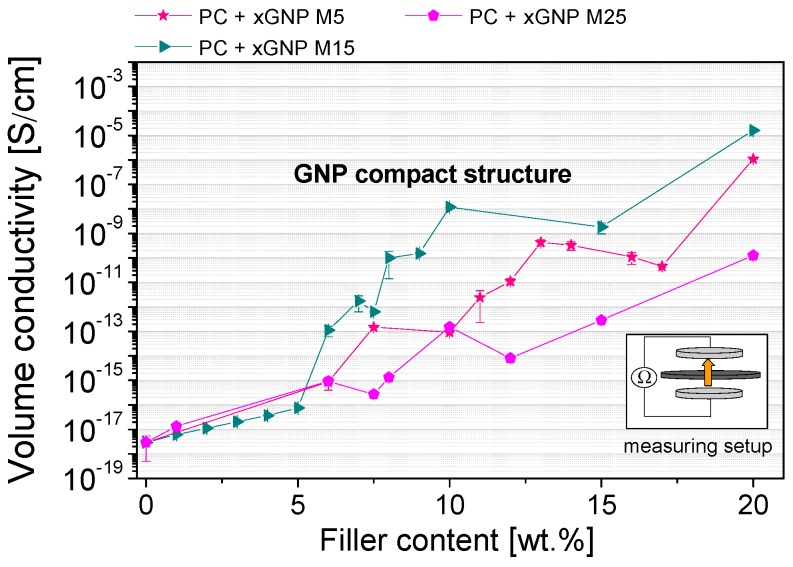
Electrical percolation behavior of compact structure GNPs with different initial particle sizes in polycarbonate (dispersion optimized processing parameters are used; 280 °C, 250 rpm, 5 min), measured through the thickness direction of compression molded plates (Keithley 8009 Resistivity Test Fixture plate configuration).

The effect of the plate aspect ratio on electrical percolation was examined using the different GNP materials of the compact structure type. According to theoretical studies the percolation threshold should increase linearly with the plate thickness and decrease in a non-linear way with plate lateral size [52]. In our work, the compact GNP materials from XG Sciences xGNP M5, M15 and M25 (Figure 1) are produced by an identical top-down method except for the final milling process which results in different lateral particle size distributions (Table 1). Figure 11 shows the influence of the initial GNP particle size on the percolation behavior. It was found that the medium initial particle size (d_50_ = 15 µm) of the xGNP M15 primary structures is favorable for a lower percolation threshold in comparison to the smaller and higher initial GNP particle sizes. The xGNP M5 powder with the lower particle size of d_50_ = 5 µm can be easily distributed by melt mixing, but due to the lower aspect ratio more material is needed for electrical percolation. For xGNP M25 with the largest plate size (d_50_ = 25 µm), the aspect ratio is certainly higher, however due to less effective exfoliation during melt mixing the numbers of GNP particles are too low to percolate at lower filler contents.

**Figure 12:**
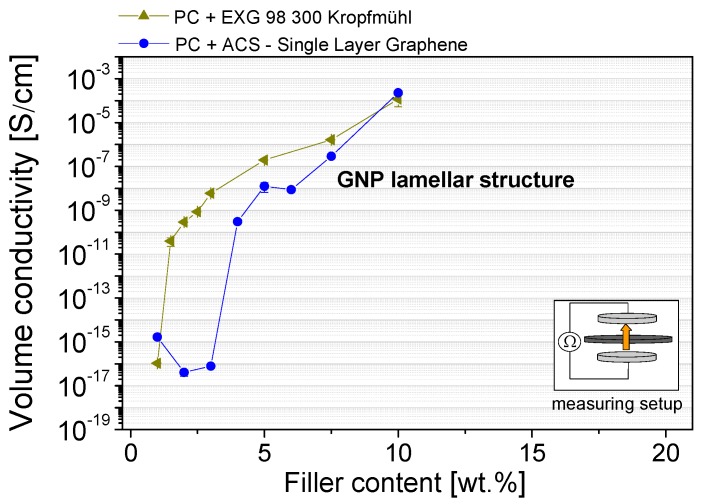
Electrical percolation behavior of different types of lamellar structure GNPs in polycarbonate (dispersion optimized processing parameters are used), measured through the thickness direction of compression molded plates (Keithley 8009 Resistivity Test Fixture plate configuration).

The influence of the aspect ratio of the plates on the percolation threshold was also studied using lamellar GNP structures (Figure 12). The percolation threshold for the ACS-Single Layer Graphene and EXG 98 300 Kropfmühl was 2 wt % and 4 wt %, respectively, whereby the initial powders show larger lamella sizes for the latter (Figure 2). Additionally, the looser structure of the lamellar EXG 98 300 Kropfmühl material, which is also characterized by a lower bulk density in comparison to the ACS-Single Layer Graphene powder (Table 1), can be infiltrated by the polymer melt more easily. As a result the distribution and dispersion of the GNP lamellas are expected to be more promoted as compared to the ACS product.

### 3.4. Mechanical Properties

**Figure 13:**
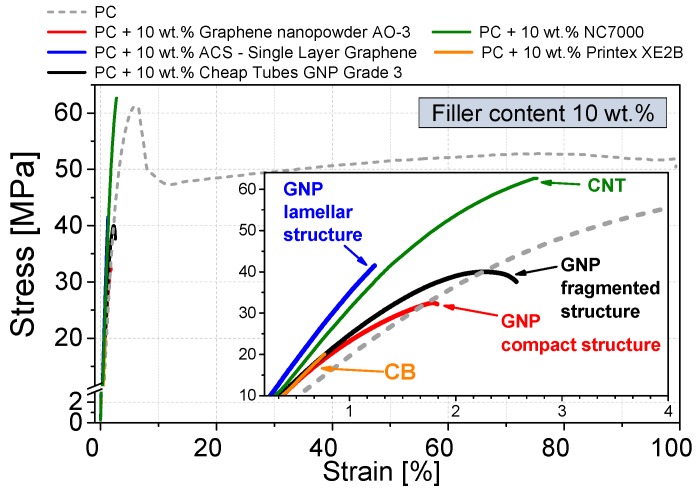
Stress–strain behavior of various commercially available carbon fillers in polycarbonate (optimized processing parameters), measured on bars punched from compression molded plates.

**Figure 14:**
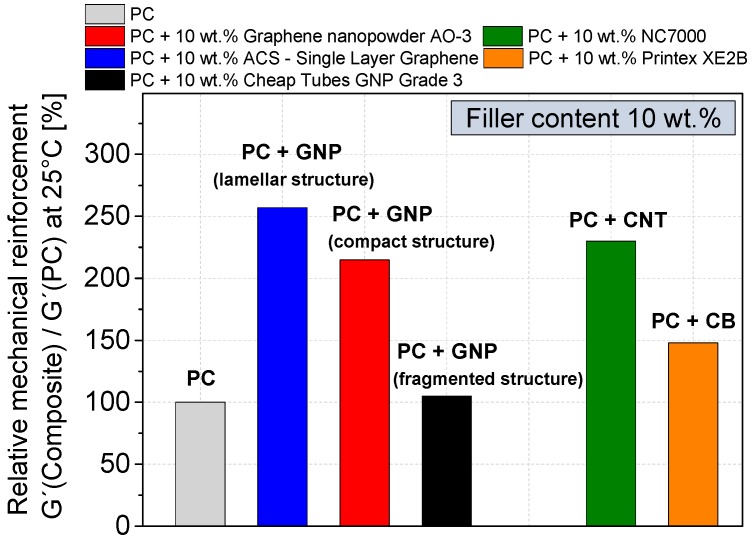
Relative shear modulus (DMTA measurements) of GNP, CNT and CB based PC composites.

The mechanical properties of the filler dispersion optimized composites (used mixing parameters see Table 3) were investigated by tensile testing and dynamic mechanical thermal analysis (Figure 13 and Figure 14, respectively). To evaluate the influence of different GNP structures, a relatively high filler content of 10 wt % was chosen. At this loading, all GNP composites are electrically percolated or in the case of the fragmented GNP (Cheap Tubes GNP Grade 3) the percolation range starts.

Adding 10 wt % of the different GNP structures affects the stress–strain behavior significantly (Figure 13). As the sample break of the filled samples occurs at very low strains, in contrast to pure PC no strain hardening behavior is visible. Similar result of strong decrease in elongation at break starting at 10 wt % filler was reported by King et al. for xGNP M5 material (compact structure type) in PC [53]. In our work, tensile strength decreases for all three GNP structures from 60 MPa of the unfilled polycarbonate to about 40 MPa. One reason for the serious reduction in tensile strength and elongation at break is the poor macrodispersion of the GNP (Figure 7 and Figure 8). The remaining GNP primary structures, in the order of several micrometers, act as stress concentrators where the material fails.

The reinforcement effect of the different carbon fillers at room temperature was measured by dynamic mechanical thermal analysis (DMTA, Figure 14). For all GNP based composites, an increase in the shear modulus can be recognized. The highest reinforcement with an increase in the shear modulus G′ by 160% was found for the lamellar structure GNP ACS-Single Layer Graphene. The compact GNP shows an increase by 115%, while the reinforcement of the fragmented structured GNP Cheap Tubes Grade 3 is negligible. The CNT reference shows in comparison to various GNP materials a higher tensile strength of 62 MPa and reinforcement of shear modulus by 130%, however at 10 wt % loading the elongation at break is also very low.

### 3.5. Thermal Properties

**Figure 15:**
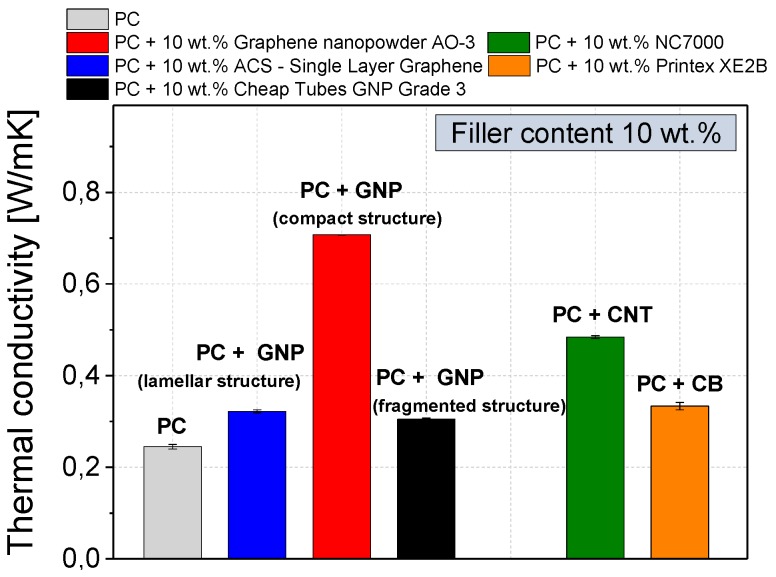
Thermal conductivity of different commercially available carbon fillers in polycarbonate (dispersion-optimized processing parameters), measured on a compression molded plates (using a HotDisc device) at a filler content of 10 wt %.

The effect of the different GNP structures on the thermal conductivity of dispersion optimized GNP composites was also determined at the filler loading of 10 wt % (Figure 15). The processed virgin polycarbonate has a very low thermal conductivity of 0.24 W∙m^−1^∙K^−1^, as is common for amorphous polymers. This value can be increased by addition of a certain amount of a highly thermal conductive material.

The addition of 10 wt % carbon black or fragmented (Cheap Tubes GNP Grade 3) or lamellar (ACS-Single Layer Graphene) GNP structure powder increased the thermal conductivity slightly by about 33%. In comparison, if 10 wt % of a compact GNP structure material (Graphene nanopowder AO-3) is used, the thermal conductivity in the corresponding composite can be increased by 196% towards 0.71 W∙m^−1^∙K^−1^. Such large differences in the thermal conductivity are caused by the respective contributions of lattice vibrations of the polymer and filler material. These vibrations are attenuated by every polymer-polymer or polymer-filler interface transition step, as described by the interfacial thermal resistance term [54]. Therefore, a more continuous filler material, such as the compact GNP material with higher aspect ratio, is favorable due to fewer interface transitions. The fragmented and lamellar GNP structure types exhibit higher surface area (Table 1), consequently more interfacial transitions per volume can be expected, which is reflected by the comparatively low thermal conductivities.

**Figure 16:**
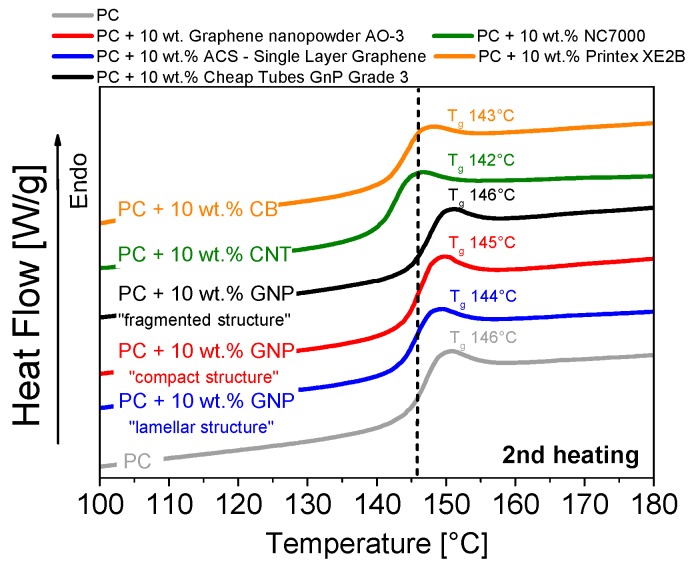
Glass transition temperature *T*_g_ (measured by DSC) of various commercially available carbon fillers in polycarbonate (dispersion-optimized processing parameters).

The calorimetric properties, e.g., the glass transition temperature *T*_g_, were determined using differential scanning calorimetry (Figure 16). The glass transition temperature of polycarbonate (146 °C) did not change upon the addition of any of the carbon fillers. This is in accordance to literature findings [32]. Hence the maximum service temperature of these composite materials, which is very interesting for potential applications, cannot be increased by the carbon filler addition. Furthermore, the thermograms of the first and second heating do not show observable evidence of any matrix crystallinity or effective nucleation effect of GNP nanoplates in this type of PC.

### 3.6. Gas Barrier Properties

**Figure 17:**
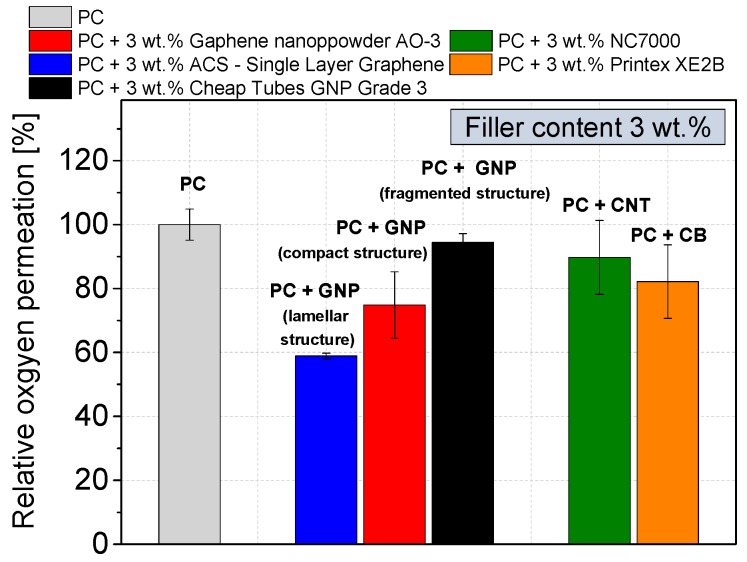
Relative oxygen permeability of commercially available carbon fillers in polycarbonate (dispersion optimized processing parameters); measured on compression molded plates.

Due to their shape, plate-like fillers are very effective at decreasing the gas permeability of a polymer. Therefore, the efficiency of the different GNP structures in terms of the best possible oxygen barrier properties at ambient temperature was verified. Here, only loadings of 3 wt % were used, otherwise a homogeneous and unperforated test sample could not be ensured. Figure 17 shows the relative oxygen permeability of the GNP composites related to that of the virgin polycarbonate (oxygen permeation coefficient of 7.3 × 10^−14^ cm^2^∙Pa^−1^∙s^−1^).

The best reduction of oxygen permeability (by 41%) was achieved using a lamellar GNP structure material (ACS- Single Layer Graphene). In contrast, the compact GNP (GNP Graphene nanopowder AO-3) reduced the gas permeation by about 25%, and the fragmented GNP (Cheap Tubes GNP Grade 3) by only 6%. The fragmented GNP structure is less effective than other carbon based fillers, such as CNT with a gas permeation reduction of 10% or CB with a reduction of 18%.

The gas permeability depends on the length of the tortuous diffusion paths which is dependent as well on the size and number as the aspect ratio of the plates [55,56]. The lamellar structure, which has the highest aspect ratio, results in the longest diffusion length as compared to the other structures. In addition, more GNP particles per volume unit of the lamellar and of the fragmented GNP at the nanoscale hinder the gas permeation to a greater extent. However, the fragmented structure has the lowest aspect ratio among the fillers. On the other hand, the size of the remaining primary GNP structures has to be considered, which is the highest in case of the lamellar structure and the lowest for the fragmented structure.

Further the different surface area values (Table 1) of the GNP materials must also be considered, since the gas diffusion path through the polymer increases with increasing aspect ratio. The evident difference of the barrier properties between the lamellar GNP and the fragmented GNP (Figure 17) can be considered as confirmation of this assumption.

## 4. Conclusions

Diverse industrial graphite nanoplate materials with different morphologies were investigated to determine their dispersion behavior by melt mixing in polycarbonate. The effect of the resulting differences in morphology on the electrical, thermal and mechanical properties of PC based composites was studied. In this investigation, we examined three clearly different GNP morphologies, which were manufactured by different production routes, so that lamellar, fragmented or compact GNP structures were formed.

The study illustrates that for GNP composite applications, the consideration of the initial GNP morphology is crucial. Depending on the morphology type, the GNP materials show different dispersion behavior during the melt mixing process and this affects the electrical, thermal and mechanical properties of the resulting composites.

In general, the dispersion evolution of the respective GNP types is similar under variation of melt temperature, screw speed or mixing time. Increased shear stresses particular reduce the lateral dimensions of the GNP primary structures, whereby the GNP aspect ratio decreases. A full exfoliation of the graphite nanoplates to individual graphene layers during the melt mixed process could not be realized. However, some exfoliated few-layer graphene sheets are visible in the TEM pictures, whereas the visible amount differs by the respective GNP morphology. The resulting states of GNP macrodispersion of the dispersion optimized composites are attributed to the different GNP morphology types, particle sizes and bulk densities. Compared to other carbon based fillers such as CNT or CB the following characteristics are obtained by the use of GNP:

(1) Among the GNP types, the lamellar GNP has the lowest electrical percolation threshold of 2 wt %, which is only slightly higher than that of CNTs with 1 wt %. However, the highest achieved electrical conductivity level (≈10^−4^ S∙cm^−1^ at 10 wt % loading) is significantly lower than for CNT or carbon black (≈10^−1^–10^−2^ S∙cm^−1^).
(2) The highest relative mechanical reinforcement was obtained when using GNP with lamellar structure showing an increase of the relative G′ value by 160% (loading 10 wt %) which is even higher than that when using CNTs (130%).
(3) The composite filled with compact GNP (10 wt %) exhibited the highest thermal conductivity of 0.71 W∙m^−1^∙K^−1^, which corresponds to an increase by 196% compared to virgin PC with only 0.24 W∙m^−1^∙K^−1^. With all other carbon based fillers a significantly lower thermal conductivity improvement was achieved.
(4) The oxygen gas permeability can be decreased by 41% by using only 3 wt % of lamellar GNP material. Due to the plate-like shape combined with a high aspect ratio, composites with this GNP type showed better oxygen barrier properties than CNT or CB based composites.

Depending on the required property profile of a final product, such as electrical, thermal, mechanical or barrier properties, the different GNP structures have advantages and disadvantages over the others and in comparison with CNTs and CB.

## Figures and Tables

**Table 1:** Specifications of the used commercial carbon filler material powders.

Material as Named by the Producer/Producer	Morphology	Average Particle Size d_50_ [µm]	Thickness/Diameter	Electrical Conductivity [S∙cm^−1^]	Specific Surface [m^2^∙g^−1^]	Bulk Density [kg∙m^−3^]
Graphene nanopowder AO-3 Graphene Supermarket^®^	compact	50 *^3^	12 nm [42]	14 *^2^	80 [42]	45 *^1^
xGNP M5 XG Sciences	compact	5 *^3^	6–8 nm [43]	34 *^2^	120–150 [43]	160 *^1^
xGNP M15 XG Sciences	compact	15 *^3^	6–8 nm [43]	35 *^2^	120–150 [43]	66 *^1^
xGNP M25 XG Sciences	compact	25 *^3^	6–8 nm [43]	22 *^2^	120–150 [43]	67 *^1^
Single Layer Graphene ACS Material^®^	lamellar	66 *^3^	1–5 layer [44]	4 *^2^	650–750 [44]	5 *^1^
EXG 98 300 Graphit Kropfmühl	lamellar	305 *^3^	-	3 *^2^	>300 [45]	1 *^1^
GNPs *^0^ Grade 3 Cheap Tubes	fragmented	2 *^3^	8 nm [46]	5 *^2^	600–750 [46]	229 *^1^
Nanocyl™ NC7000 (CNT) Nanocyl S.A	fibre	>675 [47]	Ø 9.5 nm [48]	15 *^2^	250–300 [48]	66 [47]
Printex XE2B (CB) Orion Engineered Carbons	spherical	60 *^3^	Ø 30–35 nm [49]	20 *^2^	1000 [49]	100–400 [49]

*^0^ stands for Graphene nanoplatelets; *^1^ in house measurement according to EN 1097-3; *^2^ at 30 MPa compression pressure; *^3^ in house laser diffraction measurement.

**Table 2:** Compression molding parameters for the various characterization methods.

Characterization Method	Dimension	Molding Parameters	Hot Press
electrical conductivity DC	round Ø 60 mm; thickness 0.3 mm	250 °C, 1.5 min, 50 kN	PW 40 EH
thermal conductivity	round Ø 25 mm; thickness 5 mm	250 °C, 1.5 min, 50 kN	PW 20
DMTA	45 mm × 10 mm; thickness 1 mm	250 °C, 1.5 min, 50 kN	PW 40 EH
gas permeation	round Ø 120 mm; thickness 0.2 mm	250 °C, 1.5 min, 50 kN	PW 40 EH
tensile test	round Ø 60 mm; thickness 0.5 mm	250 °C, 1.5 min, 50 kN	PW 40 EH

**Table 3:** GNP dispersion and processability optimized melt mixing parameters.

Sample composition	Mixing Time [min]	Temperature [°C]	Screw Speed [min^−1^]
PC + Graphene nanopowder AO-3	5	280	250
PC + ACS-Single Layer Graphene	5	260	150
PC + Cheap Tubes GNP Grade 3	5	260	250
